# Non-Coding RNAs as Emerging Biomarkers in HPV-Associated Cervical Precancer and Cancer: Molecular Mechanisms and Clinical Perspectives

**DOI:** 10.3390/genes17060714

**Published:** 2026-06-21

**Authors:** Matteo Terrinoni, Valerio Caputo, Michele Palisciano, Giuseppe Mascellino, Sandro Gerli, Alessandro Favilli

**Affiliations:** 1 Department of Obstetrics and Gynecology, University of Perugia, 06132 Perugia, Italy; matteo.terrinoni@unipg.it (M.T.); sandro.gerli@unipg.it (S.G.); alessandro.favilli@unipg.it (A.F.); 2 Department of Biomedicine and Prevention, University of Rome “Tor Vergata”, 00133 Rome, Italy; 3 Department of Obstetrics and Gynecology, “Alto Tevere” Hospital of Città di Castello, USL Umbria 1, 06127 Perugia, Italy; 4 Department of Life, Health and Environmental Sciences, University of L’Aquila, 67100 L’Aquila, Italy; 5 Department of Obstetrics and Gynecology, Bufalini Hospital, 47521 Cesena, Italy; michele.palisciano@live.it; 6 Dipartimento PROMISE del Policlinico di Palermo (Promozione della Salute Materno Infantile, di Medicina Interna e Specialistica G. d’Alessandro), 90127 Palermo, Italy; mascellinog@gmail.com

**Keywords:** cervical cancer, HPV, non-coding RNA, microRNA, lncRNA, circular RNA, exosomes, biomarkers, TUBORF, chemoradiotherapy resistance

## Abstract

**Background/Objectives:** Cervical cancer is mainly driven by persistent infection with high-risk human papillomaviruses (HPV), particularly HPV16 and HPV18. Despite advances in cytology, HPV-DNA testing and vaccination, challenges remain in the triage of HPV-positive individuals, prognostic stratification and prediction of treatment response. Non-coding RNAs (ncRNAs), including microRNAs, long non-coding RNAs and circular RNAs, together with host genetic factors influencing ncRNA expression and emerging lncRNA-encoded peptides, are increasingly recognized as regulators of HPV-associated carcinogenesis. This review summarizes their biological and potential clinical relevance. **Methods:** A structured literature search was conducted in PubMed and Scopus. Eligible studies included experimental, clinical, observational, genomic and translational investigations on ncRNA dysregulation, circulating or exosomal ncRNAs, treatment-response signatures, host genetic variation and lncRNA-encoded peptides in HPV-associated cervical precancer and cancer. **Results:** HPV oncoproteins can reshape host ncRNA networks through transcriptional and epigenetic mechanisms. Several miRNAs, lncRNAs and circRNAs are involved in cell-cycle control, apoptosis, senescence, epithelial–mesenchymal transition, immune regulation, DNA repair and treatment resistance. Circulating, exosomal and urinary ncRNA signatures have shown diagnostic or prognostic potential in exploratory cohorts. Specific lncRNAs, including ENSG00000267838/lnc-LENG9-5 and lncRNA-EME1, have been associated with chemoradiotherapy response and radioresistance. The lncRNA-encoded peptide TUBORF represents a novel preclinical therapeutic candidate, while genetic variation may further modulate lncRNA function in HPV-related cervical cancer. **Conclusions:** ncRNAs are promising candidates for risk stratification, non-invasive diagnosis, treatment-response prediction and therapeutic development in HPV-associated cervical disease. However, evidence remains exploratory, requiring prospective multicentre validation and standardized workflows before clinical implementation.

## 1. Introduction

Cervical cancer remains a major global health burden and is etiologically linked, in most cases, to persistent infection with high-risk human papillomaviruses (HPV), particularly HPV16 and HPV18 [1,2,3]. Although cytology-based screening, HPV-DNA testing and prophylactic vaccination have substantially reduced cervical cancer incidence in settings where they are widely implemented, important clinical and public-health gaps persist. Current screening strategies do not always provide optimal prognostic stratification for HPV-positive individuals, access to vaccination and screening remains uneven between and within countries, and many patients still present with advanced disease despite available prevention programmes [4,5].

At the molecular level, the viral oncoproteins E6 and E7 are central drivers of HPV-mediated transformation. Through functional disruption of p53 and retinoblastoma (Rb) pathways, and through interactions with PI3K–AKT, MAPK, Wnt/β-catenin and epigenetic regulatory networks, E6 and E7 reprogramme host signalling and chromatin states in ways that promote carcinogenesis and may influence response to therapy [5]. These perturbations extend to the non-coding transcriptome, including microRNAs (miRNAs), long non-coding RNAs (lncRNAs) and circular RNAs (circRNAs). In addition, some transcripts historically annotated as lncRNAs have been shown to contain short open reading frames encoding functional micropeptides, adding a further layer of regulatory complexity. Dysregulated ncRNAs can reinforce oncogenic circuits, impair tumour-suppressive networks, influence DNA repair and cell-cycle checkpoints, and modulate the tumour microenvironment. For these reasons, ncRNAs and related lncRNA-encoded peptides represent biologically plausible candidates for biomarker development and therapeutic targeting.

The relevance of these molecular insights should be interpreted within the broader clinical context of HPV-associated lower genital tract disease and its continuing need for more precise risk stratification, individualized follow-up and evidence-based integration of emerging therapeutic approaches [6,7,8,9]. Thus, molecular biomarker research is not separate from clinical practice, but rather may contribute to improving decision-making across prevention, diagnosis, treatment and surveillance.

**Figure 1:**
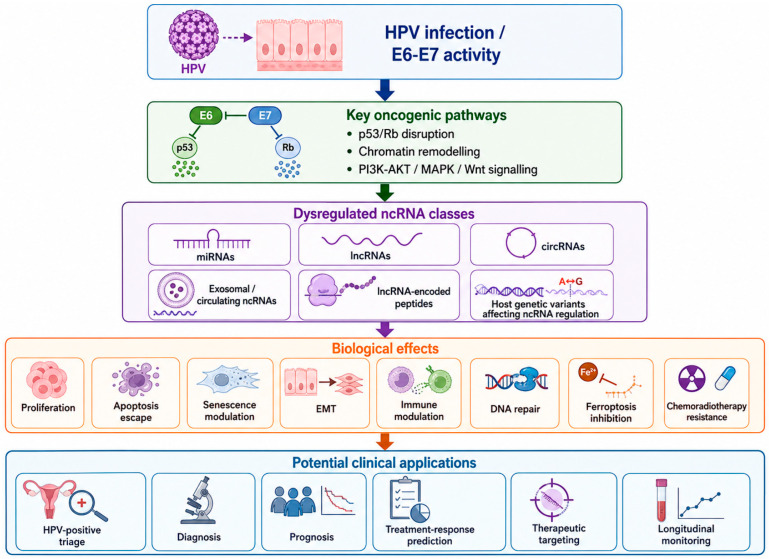
Schematic overview of ncRNA-mediated mechanisms and translational applications in HPV-associated cervical precancer and cancer.

Given the breadth and heterogeneity of the available evidence, the present article is designed as a narrative review based on a structured literature search. It aims to summarize current knowledge on ncRNA dysregulation in HPV-associated cervical precancer and cancer, with particular attention to molecular mechanisms, diagnostic and prognostic biomarker potential, treatment-response prediction and therapeutic implications. The review also discusses the main barriers to clinical translation, including methodological heterogeneity, small cohorts, limited external validation and the need for prospective multicentre studies before ncRNA-based assays or therapeutic strategies can be incorporated into routine care. A schematic overview of the proposed relationships between HPV oncogene activity, ncRNA dysregulation, biological effects and potential clinical applications is presented in Figure 1.

## 2. Materials and Methods

### 2.1. Search Strategy and Selection Criteria

This narrative review assessed following the SANRA, the Scale for the Assessment of Narrative Review Articles [10], was based on a structured literature search performed in PubMed and Scopus. The search was designed to identify relevant studies investigating non-coding RNAs and related molecular mechanisms in HPV-associated cervical disease, with particular attention to cervical intraepithelial neoplasia, cervical cancer, biomarker development and therapeutic implications. The following combinations of keywords were used: “HPV” AND “cervical cancer”; “non-coding RNA” AND “cervical cancer”; “microRNA” OR “miRNA” AND “cervical cancer”; “long non-coding RNA” OR “lncRNA” AND “cervical cancer”; “circular RNA” OR “circRNA” AND “cervical cancer”; “exosome” OR “exosomal RNA” OR “exosomal non-coding RNA” AND “cervical cancer”; “biomarker” AND “cervical cancer”; and “peptide” OR “lncRNA-encoded peptide” AND “cervical cancer”. Additional relevant articles were identified by manually screening the reference lists of selected papers. Eligible articles included original experimental studies, clinical studies, observational studies, case–control studies, cohort studies, multicentre studies, genomic association studies and relevant translational studies investigating miRNAs, lncRNAs, circRNAs, exosomal or circulating ncRNAs, and lncRNA-encoded peptides in HPV-associated cervical disease. Studies were considered if they were published in English and addressed at least one of the following aspects: molecular mechanisms, diagnostic or prognostic biomarker potential, treatment-response prediction, therapeutic resistance or potential therapeutic targeting.

Narrative reviews, systematic reviews, editorials, commentaries, non-English articles and studies not directly related to HPV-associated cervical disease or cervical cancer were excluded from the main evidence synthesis, although selected reviews were used where appropriate to provide background or contextual information. Because of the narrative nature of this review, formal PRISMA-based study selection, protocol registration and risk-of-bias assessment were not performed.

The search identified 224 records. After deduplication, 214 titles and abstracts were screened for relevance. Articles were selected based on their relevance to the review aims rather than according to a formal PRISMA selection process; therefore, no quantitative study-selection flow was used for evidence grading.

### 2.2. Data Extraction and Synthesis

For each selected study, the following information was extracted when available: study design, experimental or clinical model, biological source of ncRNA analysis, ncRNA class and specific molecule investigated, principal molecular findings, functional assays, clinical associations and proposed translational relevance. Biological sources included cervical tissue, plasma, serum, urine, circulating RNA fractions and exosomal samples.

Given the heterogeneity of study designs, biological materials, analytical platforms and reported outcomes, no quantitative meta-analysis was performed. Findings were summarized narratively and organized by ncRNA class and translational application, including miRNAs, lncRNAs, circRNAs, circulating and exosomal ncRNAs, treatment-resistance signatures, host genetic variation and lncRNA-encoded peptides. The synthesis focused on recurring molecular mechanisms, candidate biomarkers and therapeutic perspectives, while also highlighting limitations related to small cohorts, methodological variability and lack of prospective validation.

## 3. Results

### 3.1. Overview of the Evidence

The available literature includes mechanistic cell-based studies, xenograft models, transcriptomic analyses of patient tumours, liquid-biopsy studies using plasma, serum, urine and exosomal samples, candidate biomarker panels, and genomic association studies exploring the relationship between host genetic variation and ncRNA regulation.

First, HPV oncoproteins, particularly E6 and E7, can directly or indirectly alter host ncRNA expression and processing. Second, many lncRNAs appear to participate in chromatin regulation, thereby influencing tumour-suppressor gene expression. Third, circRNAs often act as competing endogenous RNAs or miRNA sponges. Fourth, circulating and exosomal ncRNAs have shown promising diagnostic or prognostic performance in small discovery cohorts, although independent validation remains limited. Finally, ncRNA-mediated regulatory axes have been implicated in therapy response, including radioresistance, cisplatin resistance and DNA-repair modulation (Table 1).

### 3.2. Viral ncRNAs and HPV-Driven Regulation of Host ncRNA Circuits

HPV infection shapes the ncRNA landscape through viral-derived transcripts and host ncRNA regulation. Viral circular transcripts, such as circE7, arise from back-splicing of the *E7* locus and have been reported to encode E7 protein, supporting a direct contribution of viral circRNAs to HPV-associated oncogenesis in tumour samples and functional models [21,22].

HPV oncoproteins can reconfigure host mitochondrial and nuclear ncRNA networks. For example, E2 has been reported to downregulate antisense mitochondrial ncRNAs, including ASncmtRNA-1 and ASncmtRNA-2, in HPV-immortalized keratinocytes and transformed cell lines. Combined *E6/E7* expression has also been associated with altered expression of sense mitochondrial ncRNAs, including SncmtRNA-2, while SncmtRNA-1 appears to support proliferation in SiHa and HeLa models [23].

HPV E6 and E7 also influence ncRNA-protein circuits through epigenetic mechanisms. E6 has been shown to increase lnc_000231 expression by impairing the H3K4 demethylase KDM5C, leading to increased H3K4me3 at the lncRNA promoter. Increased lnc_000231 then acts as a competing endogenous RNA for miR-497-5p, derepressing CCNE1 and promoting cell-cycle progression [24]. Similarly, HPV16 E7 has been implicated in interactions involving EZH2 and lncRNAs, including AC103563.8, contributing to the repression of tumour-suppressor genes such as MAL through increased promoter H3K27me3 [25]. Together, these studies indicate that HPV oncoproteins regulate ncRNA networks through both transcriptional and chromatin-mediated mechanisms.

### 3.3. MicroRNAs: Mechanistic Roles and Clinical Associations

MicroRNAs are the most extensively investigated ncRNA class in HPV-associated cervical disease. Both tumour-suppressive and oncogenic miRNAs have been described.

Among tumour-suppressive miRNAs, miR-34b has been reported to promote a senescence-associated phenotype by repressing TWIST1, increasing ROS and γH2AX, and activating a p53/p21/Lamin A/C-dependent senescence programme. Higher miR-34b expression was associated with improved overall survival in TCGA cervical squamous cell carcinoma and endocervical adenocarcinoma data, while senescent cells induced by miR-34b showed sensitivity to the senolytic agent RITA in vitro [11]. Similarly, miR-193a-3p and miR-193b-3p are downregulated during HPV-induced progression, and their restoration suppresses anchorage-independent growth by targeting multiple regulators of the PI3K–AKT pathway [12]. miR-129-5p is epigenetically silenced during transformation and inhibits anchorage-independent growth through regulation of *ACTN1* and the ELK4/c-FOS axis [26]. miR-140-5p has also been shown to target *FEN1*, reducing proliferation and migration while promoting apoptosis and G1 arrest [27].

miR-96 promotes cervical cancer cell proliferation and invasion through Akt/mTOR activation by targeting *CAV1* [28]. miR-182-3p is upregulated in cervical cancer and has been linked to repression of FLI-1, which may also be affected by promoter hypermethylation. This axis has been associated with immune-related pathway alterations, suggesting a possible role in tumour–immune interactions [29].

For example, miR-497-3p and miR-324-3p negatively regulate *HDAC8* and *HDAC6*, respectively, increasing acetylation of p53, α-tubulin and Hsp90 and suppressing epithelial–mesenchymal transition and migration [13].

From a clinical perspective, circulating and self-sample-based miRNAs have shown potential as diagnostic or triage biomarkers [30]. A genome-wide miRNA analysis of HPV-positive self-samples identified a 9-miRNA panel for CIN3 detection with an AUC of 0.78 [31,32]. However, available studies remain heterogeneous in sample source, normalization methods, patient populations and validation strategy. Thus, clinical utility requires larger prospective and externally validated studies.

### 3.4. Long Non-Coding RNAs: Chromatin Modulation, ceRNA Activity and Therapy Resistance

Long non-coding RNAs participate in cervical cancer biology through chromatin remodelling, ceRNA activity, apoptosis regulation and DNA-repair modulation [33].

MEG3 is generally reported as downregulated in cervical cancer. Its overexpression increases p53 and cleaved caspase-3 expression, inhibits proliferation and promotes apoptosis, at least partly through negative regulation of miR-21-5p [14]. In contrast, MALAT1 is frequently reported as overexpressed in HPV-positive cervical cancer and has been linked to radioresistance. MALAT1 silencing reduces clonogenic survival, promotes apoptosis, induces G2/M arrest and may modulate radiosensitivity through miR-145 sponging [15,16].

Other lncRNAs function through ceRNA mechanisms. RUSC1-AS1 acts as a competing endogenous RNA for miR-744, thereby increasing Bcl-2 expression and supporting tumour growth; its knockdown reduces tumour burden in vivo [34]. Conversely, ILF3-AS1 has been reported as downregulated in cervical cancer, and its restoration suppresses proliferation, invasion and migration while promoting apoptosis through the ILF3-AS1/miR-454-3p/PTEN axis [35].

LncRNAs have also been proposed as predictors of treatment response. ENSG00000267838/lnc-LENG9-5, was associated with progression risk and non-response to cisplatin-based chemoradiotherapy, suggesting potential relevance for treatment stratification [36]. This finding should be considered preliminary. Mechanistically targetable lncRNAs include lncRNA-EME1, which is upregulated in radioresistant cervical cancer models and enhances BRCA1 recruitment and homologous recombination. Its knockdown sensitized experimental models to radiation and PARP inhibition, supporting DNA-repair-targeted combination strategies [37].

### 3.5. Circular RNAs: Stability and ceRNA Activity

Circular RNAs are relatively stable covalently closed transcripts. In cervical cancer, they have mainly been investigated as miRNA sponges, regulators of RNA-binding proteins and candidate non-invasive biomarkers.

Among representative circRNAs, circ_0005576 is upregulated in cervical cancer and promotes tumour progression through the miR-153-3p/KIF20A axis. Its expression has been associated with advanced FIGO stage, lymph-node metastasis and worse overall survival, while circ_0005576 silencing reduces proliferation, migration and xenograft growth [17]. circWHSC1/circNSD2 may promote malignant phenotypes through the miR-532-3p/LTBP2 axis and TGF-β signalling, although cervical cancer-specific validation remains limited [38]. Another circRNA, hsa_circ_0000276, also referred to as circTRIM22, is elevated in HPV16-positive precancerous lesions and squamous cell carcinoma. It acts through a ceRNA network involving miR-154-5p and downstream targets such as CD47, LDHA, PDIA3 and SLC16A1, with reported associations with prognosis and immune infiltration. Knockdown experiments suggest effects on G1/S arrest and apoptosis [39].

Overall, circRNAs are promising biomarker candidates, but clinical translation remains limited by small cohorts, non-standardized detection methods and insufficient external validation.

### 3.6. Circulating and Exosomal ncRNAs

Circulating and exosomal ncRNAs are promising minimally invasive biomarkers for diagnosis, prognosis and treatment monitoring. Extracellular vesicles can concentrate diverse RNA species, supporting liquid-biopsy development.

A pilot study profiled plasma exosomal RNAs across multiple RNA classes in 30 cervical cancer patients and 12 controls. The authors identified a composite signature including miR-142-3p, selected mRNAs and a snoRNA set. A reduced three-marker panel composed of RGS18, SNORA12 and SNORD97 showed very high discriminatory performance in that cohort, with an AUC of 0.99 [18]. This result remains limited by the small sample size and discovery-stage design.

Exosomal RNA dynamics during chemoradiotherapy have also been explored. Post-treatment changes, including increased exosomal miR-574-3p and decreased LINC01003, together with ACOT9 mRNA changes, were associated with early death and stratified 30-month disease-specific survival in a retrospective analysis [40]. These findings suggest that longitudinal exosomal RNA profiling may have prognostic value, although validation in larger prospective cohorts is required.

Urine-based miRNA signatures have also been proposed as non-invasive diagnostic tools. Urinary miR-145-5p, miR-218-5p and miR-34a-5p showed diagnostic and prognostic potential in a cohort of cervical cancer patients and controls [30]. Plasma oncogenic miRNAs have also been investigated during chemoradiotherapy, but results across circulating miRNA studies remain inconsistent, likely because of differences in sample handling, RNA extraction, normalization strategies, disease stage and treatment timing [31].

### 3.7. ncRNAs and Therapy Resistance: Mechanistic and Predictive Evidence

Several ncRNAs have been implicated in radioresistance and cisplatin resistance. A transcriptome comparison of SiHa and cisplatin-resistant SiHa/DDP cells identified multiple differentially expressed coding and non-coding RNAs. Among them, the AC010198.2/miR-34b-3p/STC2 axis was proposed as a mechanism contributing to cisplatin resistance. Silencing AC010198.2 restored cisplatin sensitivity in vitro by reducing colony formation and increasing apoptosis [41].

ENSG00000267838/lnc-LENG9-5 was associated with non-response to chemoradiotherapy and worse disease progression, suggesting potential use as a treatment-response biomarker [36].

Radioresistance has also been linked to lncRNA-mediated DNA-repair regulation. lncRNA-EME1 enhances BRCA1 recruitment and homologous recombination, thereby promoting radioresistance. Experimental knockdown of lncRNA-EME1 increased sensitivity to radiation and PARP inhibitors in cervical cancer models [37]. In addition, miR-34b-induced senescence may create vulnerability to senolytic treatment with RITA, suggesting a preclinical pro-senescence/senolytic strategy [11].

### 3.8. Genetic Variation as a Modulator of ncRNA Expression and Disease Susceptibility

Host genetic background may influence ncRNA expression, post-transcriptional regulation and susceptibility to HPV-associated cervical disease. Although less developed than expression-based biomarker research, this area suggests that genetic variants may affect ncRNA-mediated networks [42].

A two-stage genome-wide association study identified 14q12 as a candidate susceptibility locus for cervical cancer. Functional follow-up suggested that rs225902 may act as a cis-eQTL for *FOXG1* and nearby nuclear lncRNAs, including CTD-2251F13/lnc-PRKD1-1 and CTD-2503I6/lnc-FOXG1-6 [19].

Meta-analytic evidence suggests protective associations for *MIR146A* rs2910164 and *MIR196A2* rs11614913, while *MIR499* rs3746444 does not appear to show a consistent association with cervical cancer risk [33].

Host genetic variation may add to integrative risk modelling with tumour, circulating and exosomal ncRNA profiling. However, larger and ancestrally diverse cohorts are required to identify robust genetic associations.

## 4. Discussion

This review highlights ncRNAs as interconnected regulators and candidate biomarkers in HPV-associated cervical disease. Across the available literature, miRNAs, lncRNAs, circRNAs, circulating and exosomal ncRNAs, host genetic variants affecting ncRNA regulation and recently described lncRNA-encoded peptides emerge as components of HPV-driven carcinogenesis. These alterations participate in multilayer regulatory networks involving viral oncogene activity, chromatin remodelling, DNA repair, apoptosis, epithelial–mesenchymal transition, immune modulation and treatment response [13,18,19,20,21,22,23,24,25,36,37]. From a clinical perspective, ncRNAs are relevant because they may bridge molecular mechanisms with practical needs in cervical cancer care, including triage of HPV-positive women, non-invasive disease monitoring, risk stratification and prediction of treatment response.

HPV oncoproteins reshape the host ncRNA landscape through transcriptional and epigenetic mechanisms. Beyond the established E6- and E7-mediated disruption of p53 and Rb pathways, recent findings suggest that these viral proteins interact with chromatin modifiers and ncRNA-associated regulatory circuits. Examples include the KDM5C/lnc_000231/miR-497-5p/CCNE1 axis and the HPV16 E7–AC103563.8–EZH2–MAL pathway [24,25]. Viral-derived transcripts such as circE7 and HPV-regulated mitochondrial ncRNAs further support the concept that HPV infection influences both viral and host ncRNA networks [21,23]. Thus, ncRNAs may not only reflect malignant transformation but also contribute to lesion maintenance and progression. Among ncRNA classes, miRNAs remain the most extensively characterized. Tumour-suppressive miRNAs such as miR-34b, miR-193a/b-3p, miR-129-5p and miR-140-5p appear to counteract oncogenic pathways, whereas miRNAs such as miR-96 and miR-182-3p may promote malignant phenotypes [11,12,26,27,28,29]. In addition, miR-497-3p and miR-324-3p regulate HDAC8 and HDAC6, respectively, connecting miRNA dysregulation with epigenetic and post-translational control of tumour behaviour [13]. Thus, miRNA signatures are promising diagnostic, prognostic and treatment-response biomarkers, but require external validation and assay standardization before clinical use [30,31,32]. Clinically validated miRNA panels could support triage of HPV-positive women and help identify patients requiring closer surveillance.

LncRNAs provide an additional regulatory layer through chromatin remodelling, ceRNA mechanisms, apoptosis regulation and DNA-repair modulation. Several lncRNAs, including MEG3, MALAT1, RUSC1-AS1, ILF3-AS1, lnc-LENG9-5 and lncRNA-EME1, may influence tumour biology and treatment response [14,15,16,34,35,36,37]. lncRNA-EME1 has been linked to BRCA1 recruitment and homologous recombination, suggesting a connection between lncRNA biology, DNA-repair proficiency and sensitivity to radiation or PARP inhibition [37]. Similarly, lnc-LENG9-5 has been associated with non-response to chemoradiotherapy and disease progression, supporting its potential value as a candidate predictive biomarker [36]. However, many findings derive from cell-line models or small retrospective cohorts, limiting clinical robustness.

CircRNAs are also gaining attention as stable regulators and candidate biomarkers. Molecules such as circ_0005576 and hsa_circ_0000276/circTRIM22 have been associated with tumour progression, immune-related pathways and adverse clinicopathological features [17,39]. circWHSC1/circNSD2 has also been implicated in malignant phenotypes through ceRNA-mediated signalling, although cervical cancer-specific evidence remains limited [38]. Their stability makes circRNAs attractive candidates for tissue-based and liquid-biopsy biomarker development [22]. However, cervical cancer-specific circRNA evidence remains exploratory, with limited agreement on candidates for validation.

Liquid biopsy represents one of the most clinically attractive applications of ncRNA research. If validated, circulating and exosomal ncRNA assays could complement current screening and follow-up strategies by providing repeatable, minimally invasive information on lesion progression, residual disease, recurrence risk and response to chemoradiotherapy. Some reported signatures, such as the exosomal RGS18/SNORA12/SNORD97 panel and urinary miR-145-5p/miR-218-5p/miR-34a-5p, showed encouraging diagnostic performance in discovery cohorts [18,30]. Exosomal RNA dynamics during chemoradiotherapy, including changes in miR-574-3p and LINC01003, have also been associated with early death and disease-specific survival [40]. However, these results should be interpreted cautiously, as high AUC values in small studies may reflect overfitting, cohort-specific effects or technical variability. Future studies should prioritize prospective designs, predefined endpoints, independent validation cohorts and comparison with existing clinical standards such as HPV genotyping, cytology, colposcopy and histology.

From a clinical and healthcare perspective, ncRNA-based biomarkers may be most valuable if they improve the triage of HPV-positive women, reduce unnecessary colposcopies, identify patients at higher risk of progression, or predict response to chemoradiotherapy. Any future assay must demonstrate added value over HPV genotyping, cytology, colposcopy, histopathology and established clinical risk factors. Implementation studies should also assess feasibility, turnaround time, cost, accessibility, reproducibility across laboratories and applicability across different healthcare settings. This is particularly relevant in settings with uneven vaccination and screening coverage, where non-invasive biomarkers may help optimize referral and follow-up pathways. The main potential clinical applications, current evidence level and implementation barriers of ncRNA-based approaches are summarized in Table 2.

Several reviewed studies suggest that ncRNA axes may influence response to cisplatin, radiotherapy and combined chemoradiotherapy. The AC010198.2/miR-34b-3p/STC2 axis, lnc-LENG9-5 and lncRNA-EME1 represent examples of molecules or circuits potentially relevant to treatment response [36,37,41]. This is clinically relevant because resistance to cisplatin-based chemoradiotherapy remains a major cause of treatment failure in locally advanced disease. Candidate ncRNA signatures should be tested in longitudinal cohorts with standardized treatment protocols, clearly defined response criteria and survival outcomes. In medical practice, treatment-response ncRNA signatures could help identify patients who may benefit from intensified surveillance, alternative therapeutic strategies or inclusion in trials testing radiosensitizing, DNA-repair-targeted or RNA-based approaches.

The integration of host genetic variation with ncRNA profiling may further enhance risk modelling, particularly within liquid biopsy-based approaches. The 14q12 locus, particularly rs225902, together with variants such as *MIR146A* rs2910164 and *MIR196A2* rs11614913, suggests that inherited variation may modulate ncRNA expression or function [19,33]. Genetic associations may vary by ancestry, HPV genotype and screening background. Therefore, future studies should include larger, ethnically diverse cohorts and integrate genetic background with tumour transcriptomics, epigenomics, viral features and circulating biomarkers.

LncRNA-encoded peptides represent an emerging area. Some lncRNAs contain short open reading frames encoding functional micropeptides. The identification of TUBORF as a lncRNA-encoded peptide linked to HPV E6/E7 activity, ferroptosis inhibition and malignant progression introduces a new dimension to ncRNA research [20]. This finding suggests that transcripts annotated as non-coding may contribute to cervical cancer through both RNA-mediated and peptide-mediated functions. Therapeutic claims remain preliminary until target engagement, safety and efficacy are demonstrated in appropriate models and early-phase studies.

The reviewed evidence supports the biological relevance of ncRNAs in HPV-associated cervical disease, but several limitations restrict clinical translation. Many studies rely on small cohorts, single-centre designs, retrospective analyses, heterogeneous sample types and variable laboratory methods [18,30,31,32,40]. Functional experiments are often limited to a small number of cell lines or xenograft models [11,12,13,17,24,34,37,41]. Independent validation is frequently lacking, and few studies evaluate whether ncRNA markers add clinically meaningful information beyond established tools.

This review has limitations. The heterogeneity of study design, biological source, analytical platform, HPV status definition, disease stage, endpoints and normalization strategies limited direct comparison and precluded quantitative synthesis. In addition, some evidence derives from preclinical models, single-centre cohorts or small discovery studies, reducing generalizability and potentially overestimating biomarker performance. Therefore, the conclusions should be considered hypothesis-generating and require prospective, multicentre validation.

For ncRNA-based biomarkers to enter clinical practice, future research should move from discovery to validation. Candidate signatures should be tested in prospective multicentre cohorts, with standardized pre-analytical protocols, reproducible assays, predefined clinical endpoints and adequate statistical power. Studies should also report whether ncRNA markers improve diagnostic or prognostic performance beyond HPV testing, cytology, histology, HPV genotyping and established clinical variables. For therapeutic applications, mechanistic studies should be complemented by pharmacological validation, delivery-system optimization and safety assessment, particularly for RNA-targeted, epigenetic, DNA-repair and ferroptosis-modulating strategies [11,13,20,25,37].

As a translational next step, the candidate signatures listed above should be evaluated in clinically annotated cohorts. These molecular markers may be integrated with genetic variants affecting ncRNA binding, processing or biogenesis, such as rs225902, rs2910164 and rs11614913, to identify clinically relevant regulatory circuits. Such signatures could be correlated with tumour features, disease progression and treatment response, helping to refine risk stratification and guide mechanistic studies on therapeutic actionability.

In summary, ncRNAs represent a biologically rich and clinically promising field in HPV-associated cervical disease. Their strongest current contribution is mechanistic, as they help explain how persistent HPV infection reshapes cellular regulatory networks and promotes malignant progression. Their role as clinical biomarkers is promising but not yet established, and their therapeutic potential remains largely preclinical. A rigorous translational pathway integrating molecular biology, liquid biopsy, host genetics and prospective clinical validation will be required before ncRNA-based tools can be incorporated into routine cervical cancer prevention, diagnosis or treatment.

## 5. Conclusions

Nc-RNAs provide a biologically plausible and clinically promising framework for improving risk stratification, treatment-response prediction and therapeutic innovation in HPV-associated cervical precancer and cancer.

Current evidence remains exploratory and limited by small, heterogeneous cohorts and insufficient external validation, especially for circulating and exosomal biomarkers. Thus, ncRNA-based biomarkers and therapeutic targets are not yet ready for routine clinical use. Future studies should adopt standardized workflows, prospective multicentre designs, and integrated analyses combining tumour, circulating and exosomal ncRNA profiles with HPV genotype, histopathology, genetic make-up and clinical outcomes.

Their successful translation will require rigorous validation, reproducible assays and demonstration of added clinical value beyond currently established screening, diagnostic and therapeutic tools.

## Figures and Tables

**Table 1:** Representative ncRNA-related signatures in HPV-associated cervical precancer and cancer.

Biomarker Class	Biomarker	Main Known Mechanism	Biological Source	Clinical Relevance	Reference
miRNA	miR-34b	TWIST1 repression; induction of senescence	Tissue	Prognostic; preclinical therapeutic vulnerability	[11]
miRNA	miR-193a/b-3p	PI3K–AKT pathway inhibition	Tissue	Tumour progression	[12]
miRNA	miR-497-3p/miR-324-3p	HDAC8/HDAC6 downregulation; EMT suppression	Tissue	Tumour progression	[13]
lncRNA	MEG3	p53 and apoptosis activation	Tissue	Tumour suppression	[14]
lncRNA	MALAT1	miR-145 sponging; radioresistance	Tissue	Radiotherapy response	[15,16]
circRNA	circ_0005576	miR-153-3p/KIF20A axis	Tissue	Poor prognosis	[17]
Complex RNA signature	RGS18 + SNORA12 + SNORD97	Composite exosomal diagnostic signature	Plasma exosomes	Diagnosis	[18]
Genetic variation	rs225902	cis-eQTL for *FOXG1* and nearby lncRNAs	Germline DNA	Disease susceptibility	[19]
lncRNA-encoded peptide	TUBORF	Ferroptosis inhibition	Tissue	Preclinical therapeutic target	[20]

**Table 2:** Translational readiness and current limitations of ncRNA biomarker applications.

Application	Examples	Sample Type	Potential Use	Current Evidence Level	Main Limitation
HPV-positive triage/CIN3 detection	9-miRNA self-sample panel	Cervicovaginal self-sample	Identify high-grade lesions	Discovery/validation cohort	Needs broader external validation
Non-invasive diagnosis	miR-145-5p, miR-218-5p, miR-34a-5p	Urine	Cervical cancer detection	Exploratory clinical cohort	Pre-analytical variability
Exosomal diagnosis	RGS18 + SNORA12 + SNORD97	Plasma exosomes	Cancer vs. control discrimination	Small pilot cohort	Small sample size, overfitting risk
Treatment monitoring	miR-574-3p, LINC01003, ACOT9	Plasma exosomes	Early death/survival stratification	Retrospective cohort	Needs prospective validation
Chemoradiotherapy response	ENSG00000267838/lnc-LENG9-5	Tissue/transcriptomic profile	Predict non-response	Exploratory clinical/transcriptomic evidence	Requires independent validation
Radioresistance	lncRNA-EME1	Tissue/cell models	Potential radiosensitization target	Preclinical	No clinical validation
Therapeutic targeting	TUBORF	Tissue/cell models	Ferroptosis-related target	Preclinical	Target engagement and safety unknown
Genetic risk modelling	*ENSG00000248975* rs225902, *MIR146A* rs2910164 and *MIR196A2* rs11614913	Germline DNA	Susceptibility/risk modelling	Genetic association evidence	Validation in larger cohorts of different ancestries

## Data Availability

No new data were created or analyzed in this study. Data sharing is not applicable to this article.

## Abbreviations

The following abbreviations are used in this manuscript:

AUC	area under the curve
CCNE1	cyclin E1
ceRNA	competing endogenous RNA
CIN	cervical intraepithelial neoplasia
circRNA	circular RNA
DNA	deoxyribonucleic acid
EMT	epithelial–mesenchymal transition
eQTL	expression quantitative trait locus
EZH2	enhancer of zeste homologue 2
FIGO	International Federation of Gynecology and Obstetrics
HDAC	histone deacetylase
HPV	human papillomavirus
hr-HPV	high-risk human papillomavirus
lncRNA	long non-coding RNA
miR/miRNA	microRNA
ncRNA	non-coding RNA
PARP	poly(ADP-ribose) polymerase
PRC2	polycomb repressive complex 2
Rb	retinoblastoma protein
RNA	ribonucleic acid
ROS	reactive oxygen species
SANRA	Scale for the Assessment of Narrative Review Articles
snoRNA	small nucleolar RNA
TCGA	The Cancer Genome Atlas
TGF-β	transforming growth factor beta
